# The Vectorcardiogram Characteristic and Its Predictive Value for Reduced Left Ventricular Ejection Fraction of Children with Duchenne Muscular Dystrophy

**DOI:** 10.31083/j.rcm2508309

**Published:** 2024-08-23

**Authors:** Yaru Cui, Shuran Shao, Linling Zhang, Liting Tang, Peihuan Xie, Li Wei, Hongyu Duan, Yimin Hua, Xiaotang Cai, Kaiyu Zhou, Chuan Wang

**Affiliations:** ^1^Department of Pediatric Cardiology, West China Second University Hospital, Sichuan University, 610041 Chengdu, Sichuan, China; ^2^West China Medical School of Sichuan University, 610041 Chengdu, Sichuan, China; ^3^The Cardiac Development and Early Intervention Unit, West China Institute of Women and Children's Health, West China Second University Hospital, Sichuan University, 610041 Chengdu, Sichuan, China; ^4^Key Laboratory of Birth Defects and Related Diseases of Women and Children (Sichuan University), Ministry of Education, 610041 Chengdu, Sichuan, China; ^5^Key Laboratory of Development and Diseases of Women and Children of Sichuan Province, West China Second University Hospital, Sichuan University, 610041 Chengdu, Sichuan, China; ^6^Department of Rehabilitation Medicine, West China Second University Hospital, Sichuan University, 610041 Chengdu, Sichuan, China

**Keywords:** Duchenne muscular dystrophy, vectorcardiogram, left ventricular ejection fraction, cardiac function, age

## Abstract

**Background:**

The prognosis of Duchenne muscular dystrophy (DMD) is poor once it develops to the stage of cardiac impairment. Recent studies have demonstrated that electrocardiogram (ECG), which consists of general ECG and vectorcardiogram (VCG), retains an extremely powerful role in the assessment of patients with reduced left ventricular (LV) systolic dysfunction. However, data regarding VCG recordings in DMD and its prognostic value for reduced left ventricular ejection fraction (LVEF) of DMD have never been reported. This study aims to describe the characteristics of VCG in children with DMD and to explore the predictive value of VCG for reduced LVEF in children with DMD.

**Methods:**

A total of 306 patients with a known diagnosis of DMD confirmed by the genetic test were retrospectively enrolled at our hospital between August 2018 and August 2022. This resulted in a total study group of 486 VCG recordings. Among them, 75 DMD patients who underwent cardiac magnetic resonance (CMR) later after one year follow-up were prospectively enrolled. The trend of VCG parameters of DMD patients across the different age span were compared with age-matched normal children. Concordance statistic analysis was further performed to assess the validity of VCG parameters in predicting the occurrence of reduced LVEF in patients with DMD.

**Results:**

DMD patients have a significantly higher heart rate, R waves in V1, QRS loop percentage in the right anterior quadrant in the horizontal plane (horizontal quadrant II) and QRS loop percentage in the anterior superior quadrant in the sagittal plane (sagittal quadrant IV) than normal children. Concordance statistic (C-statistic) showed an area under the curve of quadrant IV in the sagittal plane of baseline was 0.704. The receiver operating characteristic (ROC) curve shows that quadrant IV in the sagittal plane of 7.57% was the optimal cutoff with a sensitivity of 53.3% and a specificity of 88.3% for predicting reduced LVEF in DMD patients.

**Conclusions:**

Our study firstly showed that QRS loop percentage in the right anterior quadrant in the horizontal plane (horizontal quadrant II) and QRS loop percentage in the anterior superior quadrant in the sagittal plane (sagittal quadrant IV) could be abnormal in DMD boys as early as before 5 years old. Evaluation of the myocardium by VCG in the early age to predict possible cardiac systolic dysfunction may have important implications for the ongoing management of DMD boys.

## 1. Introduction

Duchenne muscular dystrophy (DMD) is a clinically common X-linked recessive myopathy, with an incidence in live male infants of approximately 1/3500–1/5000 [1, 2]. Most children with DMD begin to exhibit abnormal gait at 3–4 years of age, gradually lose their walking ability at 10–12 years and die of circulatory and respiratory failure at 18–20 years of age [3, 4]. With improvements in care and multidisciplinary treatment, the lifespan of DMD patients has been markedly prolonged, which has caused a shift in the leading cause of death in DMD patients from respiratory failure to heart failure [5]. Previous study has showed that the subendocardial dysfunction of left ventricular (LV) such as altered LV strain occurred as early as in the age of 3 years and some variants in the DMD encoding the cytoskeletal protein and dystrophin could cause a severe cardiomyopathy in the early phase [6]. Despite differences among LV dysfunctional indexes, left ventricular ejection fraction (LVEF) still remained a cornerstone of conducting therapeutic decisions that are related to myocardial performance in most clinical disease [7, 8]. Thus, it is essential to detect reduced LV systolic dysfunction in DMD patients for further improving the care and treatment of dystrophin-deficient cardiomyopathy.

Recent studies have demonstrated that electrocardiogram (ECG), which consists of general ECG and vectorcardiogram (VCG), retains an extremely powerful role in the assessment of patients with reduced LV systolic dysfunction [9]. Similarity, accumulating evidences have also demonstrated that ECG can be used to diagnose arrhythmia and preliminarily assess the scope of myocardial damage in children with suspected DMD, which provide important clues for clinicians to confirm the disease and judge prognosis [10]. However, ECG describes the cardiac signal as amplitude but not the orientation of the heart vector direction. Therefore, a mild, or even moderate degree of LV systolic dysfunction may not necessarily result in noticeable changes in the ECG. In addition, as our previous review suggested [11], almost all current studies focused on the characteristics of conventional ECG, data regarding VCG recordings in DMD patients were lacking.

VCG is the methodological elaboration of the ECG, which measures the dynamic cardiac electrical field with both the magnitude and vector direction. The VCG could improve the performance of ECG-based myocardial ischemia detection by affording temporal-spatial characteristics related to myocardial ischemia and capturing subtle changes in ST-T segment in continuous cardiac cycles [12]. Additionally, both the sagittal and frontal QRS-T angle not only have prognostic values on development of cardiovascular events but also have implications on cardiac functional performance. In addition, the spatial QRS maximum magnitude and area of QRS loop were also used to evaluate the degree of myocardial damage [13, 14, 15, 16, 17]. However, the prognostic value of VCG indices for decreased LVEF of DMD have never been reported.

Therefore, the purpose of the current study was (1) to uncover the VCG features in a large DMD population with broad age range; (2) to explore the predictive values of VCG parameters in occurrence of decreased LVEF in DMD within small age span.

## 2. Materials and Methods

### 2.1 Study Design and Population

Informed written consent was obtained from the parents of DMD patients after the nature of this study had been fully explained to them. The study was approved by the University Ethics Committee on Human Subjects at Sichuan University (2010002).

This retrospective study was conducted between August 2018 and August 2022 at our hospital. A total of 350 patients with a known diagnosis of DMD confirmed by the genetic test were initially enrolled. The subjects were excluded if presented with congenital heart disease or acquired heart disease (such as Kawasaki disease, myocarditis, rheumatic heart disease, immune diseases, hypertension, tumor chemotherapy) (n = 14). In addition, 30 boys were excluded as incomplete clinical or genetic information. Therefore, after the exclusion, 306 DMD boys were enrolled. Finally, this resulted in a total study group of 486 VCG recordings. Among them, 75 DMD patients who underwent cardiac magnetic resonance (CMR) later after one year follow-up were prospectively enrolled.

Then patients underwent routine cardiac evaluation at baseline that included electrocardiogram, chest radiography, pulmonary function testing, and Doppler echocardiography in our pediatric DMD center. And patients performed echocardiography to determine LVEF in those could not underwent CMR. Echocardiographic examinations were performed using the Vivid E9 ultrasound system (GE Healthcare, Little Chalfont, UK). Images were acquired by an experienced sonographer, following a standardized protocol. 2D views used in our study were apical four chamber (A4C) and apical two chamber (A2C) views with the subject in left lateral decubitus position. Then the LVEF was measured by the M-mode echocardiography or the Simpson biplane method. In the manual Simpson biplane method, endocardial borders were traced on end-systole and end-diastole in the apical two- and four-chamber view. End-diastole was defined at the peak of the electrocardiographic R-wave and/or 1 frame before mitral valve closure. End-systole was defined as 1 frame before mitral valve opening. Pulmonary function testing is initiated from the 5 years of age and performed every year at the ambulatory stage and every 6 months in non-ambulatory stage. Respiratory insufficiency could be considered if forced vital capacity (FVC) is less than 80% predicted.

### 2.2 Sample Groups

(1) DMD patients were stratified into five groups based on age: (<5 years old, n = 88), (5–6 years old, n = 84), (7–8 years old, n = 117), (9–12 years old, n = 157) and (≥13 years old n = 40). (2) Based on whether the LVEF was below 55% in the CMR after one year follow-up, the group categorized into normal LVEF (n = 60) and decreased LVEF group (n = 15).

Furthermore, 140 healthy boys were age-matched control subjects who had VCG recordings for routine physical examination in our hospital. Only those with normal results on ECG recordings were eligible for the study. Finally, 112 children with normal VCG recordings enrolled the study.

### 2.3 Electrocardiography

A 12-lead ECG was performed using routine ECG recordings (MedEx, MA-200, Beijing, China). ECG was defined abnormal if presenting with atrial flutter/fibrillation (AFL/AF), atrioventricular block (AVB) grades I–III, prolonged QTc >470 ms, or incomplete right bundle branch block (IRBBB). Additionally, ECG was assessed with special focus on ECG abnormalities known to be observed in DMD patients. Such as increased R-wave in V1 (>4 mm), increased R/S ratio in V1, pathological Q-waves (>0.2 mV) in inferior leads [18].

### 2.4 VCG Measurements

The computerized synthesis of the VCG from a 12-lead ECG was synthesized by the Kors transformation matrix [19]. In brief, Cardio-View vectorcardiogram workstation (V8.0.5, MedExECG-02.001.036.000.0002, Beijing, China) were used. Wilson lead system (i.e., frontal six-axis system and chest V1-V6 lead) was used to collect routine electrocardiogram for 40 seconds. Then moving the lead position to switch to the Frank lead system, and collect the patient’s electrocardiogram for 120 seconds and then the computer could save, analyze and process graph to the final data.

**Fig. 1.**
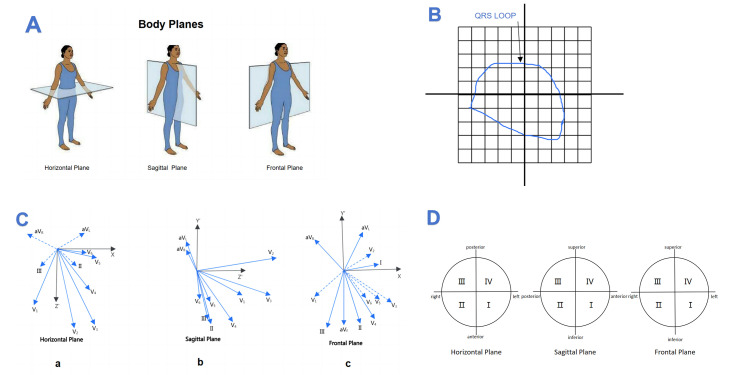
**Labeling methods for vectorcardiogram**. (A) Body plane of human. (B) A rectangle encompasses the loop in this plane and is divided into 100 subdivisions. (C,a) Leads V5 and V6 orient themselves are closer in the horizontal plane. (C,b) Leads V1, V2 and V3 orient themselves are closer in the sagittal plane. (C,c) Leads II, III and aVF orient themselves are closer in the frontal plane. (D) Clockwise method of measuring angles in the three spatial planes.

Derived VCG parameters captured for statistical analysis included the spatial QRS-T angle, spatial QRS maximum magnitude and the percentage of QRS loop in three orthogonal leads (X-lead [patient’s right to patient’s left], Y-lead [cranial-to-caudal], and Z-lead [anterior-to-posterior]), which reflects cardiac electric activity in the frontal, horizontal, and sagittal planes, were continuously monitored and analyzed (Fig. 1A). Generally, percentage of loop area (PL) is computed as the ratio of the P, QTS and T loop area to the area of the surrounding rectangle, where the rectangular area is divided into 100 equal rectangular cells, then every quadrant is divided into 25 equal rectangular cells, as shown in Fig. 1B [17]. The accurate 2-D representation of various standard leads projected by the 3D spatial vectors is shown in the Fig. 1C. Chest Leads V1-V6 lie in close vicinity of horizontal plane; Limb Leads I-AVF lie in close vicinity of frontal plane; V1-V3 lie in close vicinity of sagittal plane. In our study, in order to depict the electrical trace of ventricular depolarization in different plane, we divide every plane into four quadrants according to the study of Helm *et al*. [20], who described the clockwise method of measuring angles and defined the QRS loop tracing in different plane as frontal I-IV; horizontal I-IV; sagittal I-IV in our study (Fig. 1D).

### 2.5 Cardiac Magnetic Resonance Imaging and Measurements

CMR was acquired by cardiovascular imaging technicians with more than 3 years of experience on a 3.0T magnetic resonance imaging (MRI) scanner (Skyra, Seimens Medical Solutions, Erlangen, Germany), using an 18-channel body dedicated Coil to collect signals. Professional CMR post-processing software CVI (cvi42, version 5.13.5; Circle Cardiovascular Imaging Inc., Calgary, AB, Canada) completed a post-analysis of acquired images according to SCMR (Society of Cardiovascular Magnetic Resonance) [21] guidelines. Late gadolinium enhancement (LGE) was assessed by using T1-weighted gradient echo sequences in two-, three-, and four-chamber views and a short-axis stack contiguously covering the left ventricle. A patient with LGE presence in at least one myocardial segment was considered to be LGE positive (LGE+). If no enhancement was observed, then the subject was identified as LGE negative (LGE–). Manual correction was performed for obvious threshold errors.

### 2.6 Statistical Methods

Study results are expressed as mean ± standard deviation (SD) for continuous data and as percentages and numbers for categorical data. Continuous variables were compared using two sample *t*-test and categorical variables were compared using chi-square test by SPSS version 21 (SPSS Inc. Chicago, IL, USA). Concordance statistic (C-statistic) analysis was used to evaluate the value of VCG parameters for predicting the development of decreased LVEF. Pearson correlation analysis was used to explore associations between the parameter of R wave in V1 and VCG parameters we included in this study. All tests were 2-sided, and a *p*-value < 0.05 was considered statistically significant.

## 3. Results

### 3.1 The Trend of VCG and ECG Parameters in Different DMD Age Group

The general characteristics of the study population per age group between DMD patients and normal children are shown in the **Supplementary Tables 1–5**. Overall, the mean age was comparable. All patients have FVC than predicted 80% in patients lower than 10 years old, and one boy suffered at the age of 11.2 suffered FVC <80% but with on respiratory symptom. 2 patients were non-ambulatory whose age were 10.9 age year and 11.2 age year, respectively. The therapy with steroids was most received in boys than higher than 5 years old, and angiotensin-converting enzyme inhibitors or beta-blockers or the combination mostly used higher than 10 years old. Compared with the according separate age span of normal children, DMD patients have a significantly higher heart rate, R waves in V1, a larger QRS loop percentage in the right anterior quadrant in the horizontal plane (horizontal quadrant II) and QRS loop percentage in the anterior superior quadrant in the sagittal plane (sagittal quadrant IV) than normal children starting from small age lower than 5 years old.

In addition, as age grows, especially in patients higher than 7 years old, high R waves in V5, three orthonormal QRS maximum magnitudes, QRS loop percentage in the right inferior quadrant in the frontal plane (frontal quadrant II), QRS loop percentage in the left anterior quadrant in the horizontal plane (horizontal quadrant I) and QRS loop percentage in the anterior inferior quadrant in the sagittal plane (sagittal quadrant I) show a significantly increasing trend. And the QRS loop percentage in the left posterior quadrant in the horizontal plane (horizontal quadrant IV) and QRS loop percentage in the posterior inferior quadrant in the sagittal plane (sagittal quadrant II) show a significantly decreasing trend in patients higher than 7 years old.

### 3.2 The Correlation between R Wave in V1 and VCG Parameters

The R wave in lead V1 had a significantly positive correlation with QRS loop percentage in the right anterior quadrant in the horizontal plane (horizontal quadrant II) (r = 0.686, *p* < 0.001) and QRS loop percentage in the anterior superior quadrant in the sagittal plane (sagittal quadrant IV) (r = 0.454, *p* = 0.017). In addition, the R wave in lead V1 had a significantly negative correlation with QRS loop percentage in the right anterior quadrant in the horizontal plane (horizontal quadrant III) (r = –0.556, *p* = 0.002) and QRS loop percentage in the posterior inferior quadrant in the sagittal plane (sagittal quadrant II) (r = –0.496, *p* = 0.008) (Table 1).

**Table 1.** **Clinical correlation between R wave in V1 and vectorcardiogram profile in the DMD subjects**.

Parameters	Frontal quadrant I (%)		Frontal quadrant II (%)		Frontal quadrant III (%)		Frontal quadrant IV (%)		Horizontal quadrant I (%)		Horizontal quadrant II (%)		Horizontal quadrant III (%)		Horizontal quadrant IV (%)		Sagittal quadrant I (%)		Sagittal quadrant II (%)		Sagittal quadrant III (%)		Sagittal quadrant IV (%)	
	r	*p*	r	*p*	r	*p*	r	*p*	r	*p*	r	*p*	r	*p*	r	*p*	r	*p*	r	*p*	r	*p*	r	*p*
RV1	–0.049	0.810	0.229	0.251	–0.141	0.484	–0.026	0.898	0.230	0.248	0.686	0.001*	–0.556	0.002*	–0.299	0.130	0.498	0.008*	–0.496	0.008*	–0.414	0.032*	0.454	0.017*

DMD, Duchenne muscular dystrophy. *Significant, *p* < 0.05.

### 3.3 The Association between VCG Parameters and LVEF in the CMR Later after One Year

A total of 75 DMD boys whose LVEF were above 55% that underwent clinical CMR evaluation after one year follow-up were enrolled to analyze and showed in Table 2. 15 patients were below 55% of LVEF and grouped as decreased LVEF group (n = 15). There was no significance in the age, heart rate, weight, height as well as blood pressure between the groups. 3 boys (20.0%) loss ambulation in decreased LVEF group and 5 (8.3%) lost in normal LVEF group, and no significance was found. One patient without respiratory symptom has a lower FVC than predicted 80% and 2 patients have scoliosis in the normal LVEF group, and none in the decreased LVEF group. There was no significant difference in the use of steroid or cardiac treatment between two groups.

**Table 2.** **ECG and VCG parameters between normal LVEF and declined LVEF patient groups in DMD**.

		Declined LVEF (n = 15)	Normal LVEF (n = 60)	*p* value
Age (years)		10.64 ± 2.50	10.16 ± 2.35	0.486
Heart rate (bpm)		83.33 ± 14.12	90.25 ± 13.55	0.084
Height (cm)		130.20 ± 17.86	125.03 ± 10.58	0.298
Weight (kg)		30.61 ± 7.70	29.16 ± 13.02	0.683
Systolic blood pressure (mmHg)		103.93 ± 7.43	102.83 ± 9.12	0.667
Diastolic blood pressure (mmHg)		64.93 ± 6.07	64.05 ± 11.48	0.686
Scoliosis, n (%)		0, (0.0%)	2, (1.7%)	0.638
Loss of ambulation, n (%)		3, (20.0%)	5, (8.3%)	0.193
Respiratory Function				
	FVC% <80%, n (%)	0, (0.0%)	1, (1.7%)	0.638
Steriod Treatment				
	Corticosteroids, n (%)	14, (93.3%)	55, (91.2%)	0.655
	Time to initiate steriods (years)	7.27 ± 2.92	7.28 ± 2.70	0.991
	Time from initiating steriods (months)	27.60 ± 23.84	29.30 ± 22.91	0.799
Cardiac Treatment				
	ACE inhibitor, n (%)	3, (20.0%)	8, (13.3%)	0.382
	b-blocker, n (%)	3, (20.0%)	7, (11.7%)	0.317
	ACE inhibitor+b-blocker, n (%)	3, (20.0%)	4, (6.7%)	0.138
ECG parameters				
	P-wave axis (°)	49.73 ± 21.49	49.15 ± 24.40	0.933
	QRS-wave axis (°)	44.73 ± 14.71	49.65 ± 19.79	0.371
	T -wave axis (°)	79.60 ± 41.66	65.40 ± 21.12	0.066
	QTc (ms)	420.80 ± 14.54	417.28 ± 17.00	0.762
	P wave amplitude	0.10 ± 0.05	0.10 ± 0.04	0.565
	RV1 in amplitude	1.54 ± 0.91	1.27 ± 0.52	0.471
	RV5 in amplitude	2.18 ± 1.10	2.32 ± 0.71	0.066
	SV1 in amplitude	0.82 ± 0.65	1.04 ± 0.52	0.266
	R/S ratio in lead V1	1.62 ± 1.62	1.92 ± 1.68	0.726
VCG parameters				
	Frontal QRS maximum magnitude	5.30 ± 3.92	5.15 ± 2.52	0.860
	Horizal QRS maximum magnitude	4.55 ± 2.26	4.82 ± 1.84	0.629
	Sagittal QRS maximum magnitude	4.91 ± 2.12	6.27 ± 2.67	0.073
	Frontal quadrant I (%)	72.11 ± 19.49	72.84 ± 17.95	0.889
	Frontal quadrant II (%)	10.25 ± 8.25	12.51 ± 10.60	0.445
	Frontal quadrant III (%)	12.44 ± 15.41	13.37 ± 13.44	0.818
	Frontal quadrant IV (%)	5.20 ± 8.23	1.48 ± 3.87	0.108
	Horizontal quadrant I (%)	46.00 ± 22.35	40.27 ± 17.79	0.293
	Horizontal quadrant II (%)	9.06 ± 8.61	8.26 ± 6.60	0.694
	Horizontal quadrant III (%)	5.12 ± 5.49	5.63 ± 4.67	0.718
	Horizontal quadrant IV (%)	39.81 ± 23.35	45.76 ± 20.84	0.337
	Sagittal quadrant I (%)	40.34 ± 17.00	43.59 ± 17.85	0.527
	Sagittal quadrant II (%)	36.85 ± 25.96	46.47 ± 19.54	0.116
	Sagittal quadrant III (%)	12.73 ± 24.64	6.03 ± 6.63	0.314
	Sagittal quadrant IV (%)	10.33 ± 10.62	3.64 ± 4.01	0.030*
	Frontal QRS-T angle (°)	20.27 ± 38.61	11.28 ± 10.60	0.386
	Horizontal QRS-T angle (°)	23.67 ± 17.85	32.28 ± 28.07	0.262
	Sagittal QRS-T angle (°)	38.27 ± 34.71	57.98 ± 58.74	0.218

The data are presented as the mean ± standard deviation (SD) for continuous variables and as the percentage for the categorical variables. Frontal quadrant I, QRS loop percentage in the left inferior quadrant in the frontal plane; Frontal quadrant II, QRS loop percentage in the right inferior quadrant in the frontal plane; Frontal quadrant III, QRS loop percentage in the right superior quadrant in the frontal plane; Frontal quadrant IV, QRS loop percentage in the left superior quadrant in the frontal plane; Horizontal quadrant I, QRS loop percentage in the left anterior quadrant in the horizontal plane; Horizontal quadrant II, QRS loop percentage in the right anterior quadrant in the horizontal plane; Horizontal quadrant III, QRS loop percentage in the right anterior quadrant in the horizontal plane; Horizontal quadrant IV, QRS loop percentage in the left anterior quadrant in the horizontal plane; Sagittal quadrant I, QRS loop percentage in the anterior inferior quadrant in the sagittal plane; Sagittal quadrant II, QRS loop percentage in the posterior inferior quadrant in the sagittal plane; Sagittal quadrant III, QRS loop percentage in the posterior superior quadrant in the sagittal plane; Sagittal quadrant IV, QRS loop percentage in the anterior superior quadrant in the sagittal plane. *Significant, *p* < 0.05. DMD, Duchenne muscular dystrophy; ECG, electrocardiogram; VCG, vectorcardiogram; LVEF, left ventricular ejection fraction; FVC, forced vital capacity; ACE, angiotensin converting enzyme.

In addition, R wave in V1 or R/S ratio in V1 did not show a significant difference between the LVEF <55% and LVEF >55% groups. Of the patients with decreased LVEF, only the significantly higher QRS loop percentage in the anterior superior quadrant in the sagittal plane (sagittal quadrant IV) was noticed (*p* < 0.05). There was no significant difference in terms of positive LGE or not, age starting steroids, the steroids time, and other vectors. In addition, the comparison between positive LGE+ and negative LGE– in the CMR later after one year was also compared. A total of 61 DMD boys whose LGE were negative that underwent clinical CMR evaluation after one year follow-up were enrolled to analyze and showed in the **Supplementary Table 6**. 17 patients got positive LGE and 44 patients are negative LGE in the CMR later after one year. The ECG and VCG parameters were compared between positive LGE+ and negative LGE– group, no significant difference was found.

**Fig. 2.**
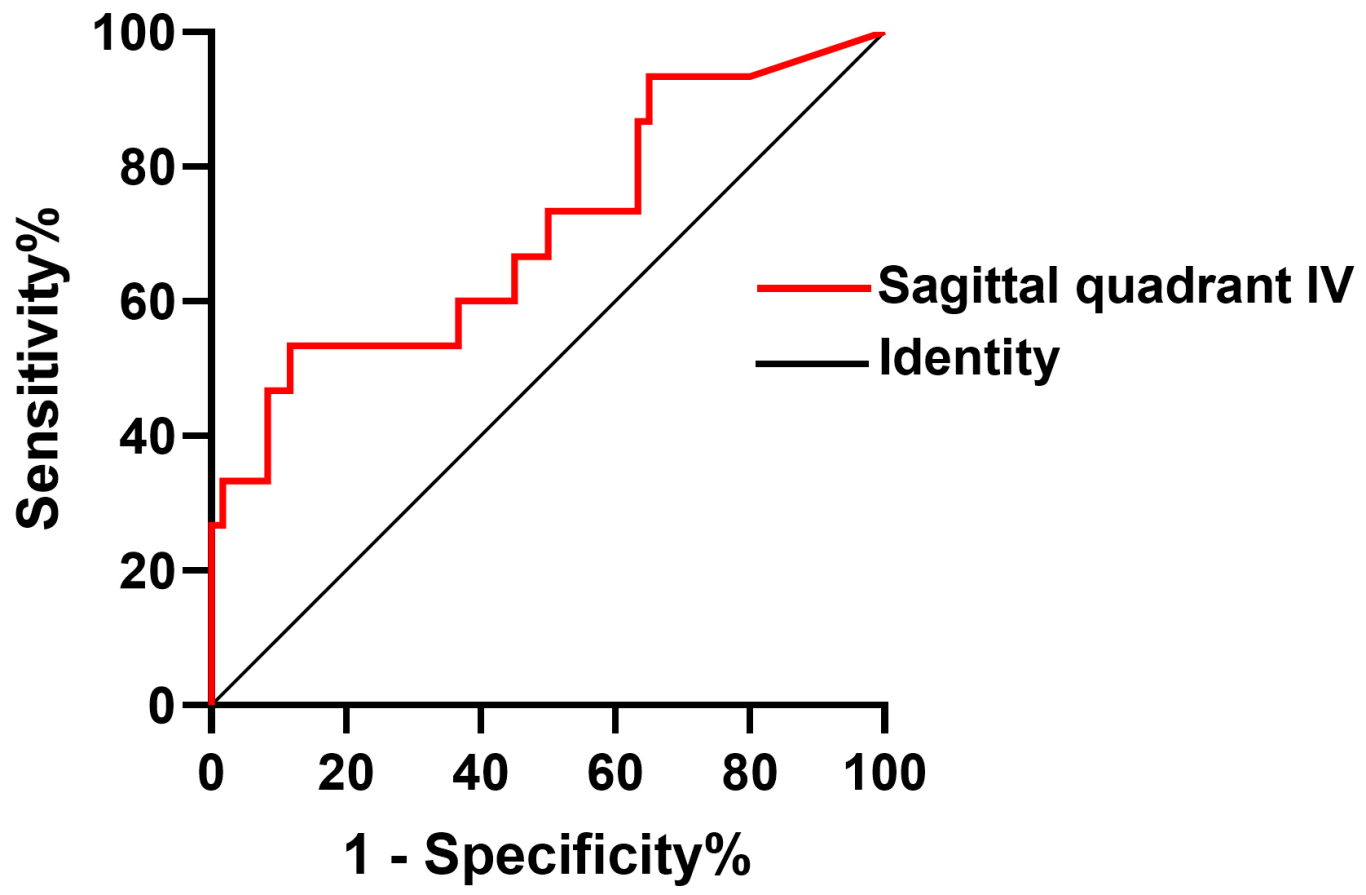
**The validity of anterior superior quadrant IV in the sagittal plane in predicting decreased LVEF in patients with DMD was assessed using C statistic**. DMD, Duchenne muscular dystrophy; LVEF, left ventricular ejection fraction.

The validity of the quadrant IV (anterior superior quadrant) in the sagittal plane in predicting decreased LVEF in patients with DMD was assessed using C-statistic. C-statistic showed an area under the curve of quadrant IV (an-terior superior quadrant) in the sagittal plane of baseline was 0.704. The receiver operating characteristic (ROC) curve shows that quadrant IV in the sagittal plane of 7.57% was the optimal cutoff with a sensitivity of 53.3% and a specificity of 88.3% for predicting decreased LVEF in DMD patients (Table 3 and Fig. 2).

**Table 3.** **The OR, sensitivity and specificity of the cutoff of QRS loop percentage in the anterior superior quadrant in the sagittal plane for predicting cardiomyopathy in DMD patients**.

Diagnostic test	Gold standard			Sen	Spe	PPV	NPV	Diagnostic accuracy	OR (95% CI)	*p*
Sagittal quadrant IV ≥7.57%	positive	8	7	0.53	0.88	0.53	0.88	0.704	8.65 (2.40–31.27)	0.001*
	negative	7	53							

CI, confidence interval; OR, odds ratio; Sen, sensitivity; Spe, specificity; PPV, positive predictive value; NPV, negative predictive value. *Significant, *p* < 0.05.

## 4. Discussion

To our knowledge, this is the first study to describe the VCG recordings in a large population with a wide age range in DMD boys around the world. Among 486 VCGs, we comprehensively analyzed the trend of VCG parameters of DMD patients compared with age-matched children across the different age span. The result shows that DMD patients already have marked abnormal right ventricular changes, which manifested as a higher R wave in lead V1, higher QRS loop percentage in the right anterior quadrant in the horizontal plane as well as QRS loop percentage in the superior anterior quadrant in the sagittal plane in VCG than normal children as early as before 5 years old.

Given the nature of progressive cardiac muscle damage of DMD, ECG abnormalities may be an early manifestation of dystrophin deficiency in contracting and/or electrically active cardiomyocytes [11]. In the study of James [10], who enrolled seventy-eight DMD patients who are less than 6 years of age, the result showed that 22% demonstrating R >98th percentile in lead V1. In the study of Girija [22], who enrolled ECGs from 252 patients with DMD (2–21 years) and ECGs from 151 age-matched healthy controls, the result found that taller R wave in V1 were significantly seen across all age group of DMD in comparison to controls. In another cross-sectional study including patients ranging in 5 to 11 years and found that 64% have a tall R wave over V1 with an abnormal R/S ratio [23]. The above findings were similar with our study that higher R wave on V1 get noticed starting from small age group. It is true that distinctive ECG pattern associated with DMD results from multifocal degenerative changes involving myocardium, predominantly the posterobasal region of the left ventricle and the posterior papillary muscle at the early stage of the disease [24]. With the increasing age of DMD boys, whose progressive cardiac dysfunction such as early LV fibrosis, LV enlargement, increased LV diastolic function and the scoliosis, lower respiratory function as well as secondary pulmonary hypertension due to hypohyoxemia would make the right ventricle be secondary hypertrophy [25]. Therefore, considering the concept that ECG abnormalities predate the development of overt hypertrophy or damaged function, the higher RV1 might be alerted that possible earlier complication with the diagnosis of secondary right ventricle hypertrophy (RVH) and involved LV dysfunction than we anticipated, and thus when the ECG of RVH path appear in routine follow-up and closer monitor is needed.

Furthermore, the association of VCG signals has never been explored with LV systolic dysfunction characterized with LVEF. We firstly prospectively enrolled 75 DMD patients with normal LVEF and explored the possible VCG risk factors of rapid deterioration of LVEF in those patients in 1 year follow up CMR. The major finding of this study is that in DMD patients, a higher QRS loop percentage in the superior anterior quadrant in the sagittal plane was significantly independently correlated with the reduced LVEF, with 53.3% and specificity of 88.3% for detecting abnormality associated with LVEF in boys with DMD, and most importantly, R wave in V1 or R/S ratio in V1 did not show a significant predictive ability between the LVEF <55% and LVEF >55% groups, which suggest that VCG might be more sensitive when evaluating LVEF. Therefore, the present study added new finding that higher QRS loop percentage in the superior anterior quadrant in the sagittal plane could be not specific but might be early markers of cardiac dysfunction.

In addition, our results firstly demonstrated that cardiac injury-induced early changes can be reflected in the VCG before the age of 5 years, and this abnormal performance might significantly develop with an increasing age. Overlapped evidence suggest that myocardial damage might be slowly progressive in small age group of DMD [10]. However, for these small age span, most were treated only with steroids, which could not prevent progression of the cardiac impairment. Previous evidence-based studies providing that angiotensin converting enzyme inhibitors (ACEI), angiotensin receptor blockers, beta-blockers and/or aldosterone antagonists might improve or preserve left ventricular systolic function and may delay the progression of cardiomyopathy [26, 27, 28, 29, 30, 31]. Actually, several types of treatment such as ACEI, beta-blockers, aldosterone antagonists, combination ACEI + beta-blockers, and aldosterone antagonists plus ACEI + beta-blockers treatments, for DMD-associated cardiac dysfunction are now available for exploring the effectiveness on LV dysfunction [32]. Though it is uncertain whether it is preferable to prophylactically treat asymptomatic patients with normal or nearly normal LV function, some of studies have reported that cohorts with younger patients with reduced LVEF had the most cardiac improvement following therapy [27, 31]. However, lack of standardization such as medication, dose, duration of treatment, differing patient ages, symptomatology, cohort size, study duration as well as baseline heart function limits the comparability of the studies and complicates assessment of the primary cardiac therapies under investigation. In addition, the newly demonstrated cardiac protective drug derived from adult such as sacubitril/valsartan, dapagliflozin as well as ivabradine have been also suggested might be effective in children with heart failure or cardiomyopathy [33, 34, 35], however, larger studies are needed to evaluate safety and efficacy of these drugs in this population. Most importantly, though the above-mentioned cardiac therapy might do suggest effective for improving the quality of life in children with symptomatic patients or in reduced LVEF, considering the pathogenesis of DMD, therapies to restore or augment dystrophin, as well as therapies that act downstream of dystrophin, may be more promising options for preserving cardiac function than standard heart failure drugs [32].

Until now, most diagnostic standard of cardiac dysfunction was relied on CMR, the price and risk of sedation as well as age span limited the small age group to perform it. And since the VCG represents magnitude, direction and the polarity of the instantaneous cardiomyocyte, the more sensitive and probable that VCG might be to show and to find cardiac abnormal demonstration. Therefore, the easily performed, good price as well as no-limited age of VCG make it possible to evaluate the cardiac function of small age group. Therefore, a larger and prospective cohort on the effects of the cardiac therapy in small age span with preserved left ventricular function is expected to explore in the future.

## 5. Study Limitations

The strengths of this study were its prospective design and relatively large sample size. However, the present study has several limitations. Firstly, this study was performed at a single institution, which could be seen as a limitation or a strength as it facilitated VCG interpretation. Secondly, since one of the purposes was to define the natural history of electrical evolution in the DMD population; future studies should focus on electrical correlations with different drug intervention and genotype-phenotype correlations in a large cohort. In addition, though some of VCG morphology features have been shown to be sensitive and specific for possible cardiac disease in this study, clinically promising and widespread study of VCG measurements has been limited because they require additional computer processing and specialized software, which can be time consuming to develop and test because variations in filtering, signal baseline definition, and signal processing preclude the direct comparison of measurements. Nevertheless, the artificial intelligence VCG algorithms as well as innovative, mobile-ECG technology have made electrical data more convenient. A larger, prospective study is needed to validate the clinical importance of these VCG morphology descriptors. Despite these limitations, this study is based on the establishment of relatively large sample prospective study cohort, prospectively combined with VCG and CMR technology to systematically evaluate cardiac function in children with DMD, analyzed the relationship between electrical abnormalities and cardiac function under different age spans, explore the 3D-electrical indicators that could change before the DMD associated cardiac disease.

## 6. Conclusions

Our study firstly showed that QRS loop percentage in the superior anterior quadrant in the sagittal plane and QRS loop percentage in the superior anterior quadrant in the sagittal plane could be abnormal in DMD boys as early as before 5 years old. In addition, the value of QRS loop percentage in the superior anterior quadrant in the sagittal plane in predicting declined LVEF were also firstly reported and depicted. Evaluation of the myocardium by VCG in early age to predict the presence of possible cardiac systolic dysfunction may have important implications for the ongoing management of DMD boys.

## Data Availability

Data generated or analyzed during this study are included in this published article.

## Abbreviations

DMD, Duchenne muscular dystrophy; ECG, electrocardiogram; CMR, cardiac magnetic resonance; VCG, vectorcardiogram; LGE, late gadolinium enhancement; LGE+, late gadolinium enhancement positive; LGE–, late gadolinium enhancement negative; ROC, receiver operating characteristic; LVEF, left ventricular ejection fraction; RVH, right ventricular hypertrophy; LV, left ventricular; RV, right ventricular; MRI, magnetic resonance imaging; ACEI, angiotensin converting enzyme inhibitors.

## Supplementary Material

Supplementary material associated with this article can be found, in the online version, at https://doi.org/10.31083/j.rcm2508309.
