# Supplementary material for: The Vectorcardiogram Characteristic and Its Predictive Value for Reduced Left Ventricular Ejection Fraction of Children with Duchenne Muscular Dystrophy
Source: Rev Cardiovasc Med. 2024 Aug 23;25(8):309. doi: 10.31083/j.rcm2508309 (PMC11367004; doi:10.31083/j.rcm2508309)
Supplement: Supplementary file 1 [file 2153-8174-25-8-309-s1.docx]

Supplementary table 1. The difference of VCG findings between normal children and DMD patients aged smaller than 5 years old.

|  | Normal children (n=28) | DMD patients (n=88) | P value |
| --- | --- | --- | --- |
| Age(years) | 3.82±0.73 | 4.10±0.73 | 0.108 |
| Heart rate (bpm) | 93.68±8.99 | 101.56±13.55 | 0.011* |
| Scoliosis, n (%) | 0, (0%) | 0, (0%) | - |
| Loss of ambulation, n (%) | 0, (0%) | 0, (0%) | - |
| FVC%<80%, n (%) | 0, (0%) | 0, (0%) | - |
| Corticosteroids, n (%) | 0, (0%) | 0, (0%) | - |
| Cardiac Treatment | 0, (0%) | 0, (0%) | - |
| **ECG parameters** |  |  |  |
| P-wave axis(°) | 37.95±15.91 | 42.43±18.06 | 0.290 |
| QRS-wave axis(°) | 37.32±12.11 | 61.42±21.16 | <0.001* |
| T -wave axis(°) | 71.82±18.63 | 35.78±14.65 | <0.001* |
| QT(ms) | 333.09±20.64 | 320.14±26.09 | 0.033* |
| QTc(ms) | 414.86±16.16 | 413.72±18.81 | 0.793 |
| P wave amplitude | 0.08±0.04 | 0.97±8.31 | 0.986 |
| RV1 amplitude | 0.68±0.39 | 1.47±0.58 | <0.001* |
| RV5 amplitude | 1.58±0.50 | 1.82±0.63 | 0.095 |
| SV1 amplitude | 0.94±0.53 | 0.96±0.55 | 0.887 |
| R/S ratio in lead V1 | 0.86±0.58 | 2.97±7.23 | 0.142 |
| RV5+SV1 amplitude | 2.52±0.87 | 2.78±0.98 | 0.252 |
| Pathological Q waves in lead II | 0, (0%) | 1/88(1.1%) | - |
| **VCG parameters** |  |  |  |
| Frontal QRS Maximum Magnitude | 3.65±1.42 | 5.02±10.85 | 0.557 |
| Horizal QRS Maximum Magnitude | 3.79±1.62 | 3.46±1.14 | 0.274 |
| Sagittal QRS Maximum Magnitude | 4.57±2.10 | 3.72±1.56 | 0.035* |
| Frontal quadrant I (%) | 77.10±11.50 | 72.05±19.21 | 0.241 |
| Frontal quadrant II (%) | 10.77±8.72 | 11.28±9.80 | 0.872 |
| Frontal quadrant III (%) | 9.68±12.20 | 13.63±12.83 | 0.195 |
| Frontal quadrant IV (%) | 2.44±5.09 | 3.29±10.81 | 0.719 |
| Horizontal quadrant I (%) | 40.05±20.13 | 45.83±20.85 | 0.245 |
| Horizontal quadrant II (%) | 3.09±4.51 | 8.99±6.86 | <0.001* |
| Horizontal quadrant III (%) | 8.37±7.82 | 6.26±5.25 | 0.132 |
| Horizontal quadrant IV (%) | 48.49±20.61 | 38.74±20.52 | 0.049* |
| Sagittal quadrant I (%) | 32.49±15.32 | 47.26±20.43 | 0.002* |
| Sagittal quadrant II (%) | 55.16±23.76 | 39.16±20.58 | 0.002* |
| Sagittal quadrant III (%) | 8.11±15.54 | 6.90±10.95 | 0.664 |
| Sagittal quadrant IV (%) | 4.24±5.76 | 6.64±5.09 | 0.056 |
| Frontal QRS-T angle (°) | 14.23±23.64 | 16.49±33.24 | 0.764 |
| Horizontal QRS-T angle (°) | 28.55±21.47 | 44.18±40.58 | 0.016* |
| Sagittal QRS-T angle (°) | 57.41±64.43 | 74.38±69.58 | 0.302 |

The data are presented as the mean ± SD for continuous variables and as the percentage for the categorical variables.

Frontal quadrant I, QRS loop percentage in the left inferior quadrant in the frontal plane; Frontal quadrant II, QRS loop percentage in the right inferior quadrant in the frontal plane; Frontal quadrant III, QRS loop percentage in the right superior quadrant in the frontal plane; Frontal quadrant IV, QRS loop percentage in the left superior quadrant in the frontal plane;

Horizontal quadrant I, QRS loop percentage in the left anterior quadrant in the horizontal plane; Horizontal quadrant II, QRS loop percentage in the right anterior quadrant in the horizontal plane; Horizontal quadrant III, QRS loop percentage in the right anterior quadrant in the horizontal plane; Horizontal quadrant IV, QRS loop percentage in the left anterior quadrant in the horizontal plane;

Sagittal quadrant I, QRS loop percentage in the anterior inferior quadrant in the sagittal plane; Sagittal quadrant II, QRS loop percentage in the posterior inferior quadrant in the sagittal plane; Sagittal quadrant III, QRS loop percentage in the posterior superior quadrant in the sagittal plane; Sagittal quadrant IV, QRS loop percentage in the anterior superior quadrant in the sagittal plane;

*significant, p<0.05

Supplementary table 2. The difference of VCG findings between normal children and DMD patients aged from 5 to 6 years old.

|  | Normal children (n=18) | DMD patients (n=84) | P value |
| --- | --- | --- | --- |
| Age(years) | 5.95±0.62 | 6.01±0.58 | 0.691 |
| Heart rate (bpm) | 81.47±13.75 | 91.56±10.43 | 0.001* |
| Scoliosis, n (%) | 0, (0%) | 0, (0%) | - |
| Loss of ambulation, n (%) | 0, (0%) | 0, (0%) | - |
| FVC%<80%, n (%) | 0, (0%) | 0, (0%) | - |
| Corticosteroids, n (%) | 0, (0%) | 80, (95.2%) | - |
| Cardiac Treatment | 0, (0%) | 0, (0%) | - |
| **ECG parameters** |  |  |  |
| P-wave axis(°) | 38.74±19.07 | 41.26±25.58 | 0.686 |
| QRS-wave axis(°) | 40.00±12.71 | 65.7±23.50 | <0.001* |
| T -wave axis(°) | 66.58±17.29 | 41.24±17.06 | <0.001* |
| QT(ms) | 353.68±32.05 | 337.51±22.79 | 0.011* |
| QTc(ms) | 407.84±21.71 | 415.18±20.74 | 0.170 |
| P wave amplitude | 0.09±0.05 | 0.51±3.88 | 0.636 |
| RV1 in amplitude | 0.68±0.28 | 1..62±0.85 | <0.001* |
| RV5 in amplitude | 1.59±0.37 | 2.20±0.82 | 0.002* |
| SV1 in amplitude | 1.05±0.57 | 1.04±0.62 | 0.989 |
| R/S ratio in lead V1 | 0.82±0.43 | 2.19±2.11 | 0.031* |
| RV5+SV1 amplitude | 2.64±0.71 | 3.24±1.19 | 0.037* |
| Pathological Q waves in lead II | 0, (0%) | 0, (0%) | - |
| **VCG parameters** |  |  |  |
| Frontal QRS Maximum Magnitude | 3.37±1.22 | 4.35±1.56 | 0.012* |
| Horizal QRS Maximum Magnitude | 3.51±1.22 | 4.02±1.22 | 0.104 |
| Sagittal QRS Maximum Magnitude | 4.14±1.90 | 4.74±1.90 | 0.219 |
| Frontal quadrant I (%) | 82.73±10.67 | 73.20±19.19 | 0.039* |
| Frontal quadrant II (%) | 6.41±6.78 | 11.58±12.20 | 0.078 |
| Frontal quadrant III (%) | 5.13±5.41 | 14.08±14.05 | 0.008* |
| Frontal quadrant IV (%) | 5.73±9.31 | 1.16±3.64 | 0.001* |
| Horizontal quadrant I (%) | 40.94±18.63 | 39.10±16.83 | 0.674 |
| Horizontal quadrant II (%) | 3.03±4.37 | 8.73±6.70 | 0.001* |
| Horizontal quadrant III (%) | 4.54±3.79 | 5.42±5.94 | 0.539 |
| Horizontal quadrant IV (%) | 51.53±18.37 | 46.76±17.50 | 0.290 |
| Sagittal quadrant I (%) | 42.49±19.99 | 42.78±15.38 | 0.946 |
| Sagittal quadrant II (%) | 46.62±24.33 | 46.07±17.04 | 0.909 |
| Sagittal quadrant III (%) | 6.57±6.93 | 6.63±10.08 | 0.981 |
| Sagittal quadrant IV (%) | 2.44±3.18 | 5.36±5.48 | 0.028* |
| Frontal QRS-T angle (°) | 8.02±8.06 | 11.00±15.98 | 0.432 |
| Horizontal QRS-T angle (°) | 22.12±17.15 | 28.14±31.22 | 0.419 |
| Sagittal QRS-T angle (°) | 47.02±51.69 | 60.88±68.16 | 0.407 |

The data are presented as the mean ± SD for continuous variables and as the percentage for the categorical variables.

Frontal quadrant I, QRS loop percentage in the left inferior quadrant in the frontal plane; Frontal quadrant II, QRS loop percentage in the right inferior quadrant in the frontal plane; Frontal quadrant III, QRS loop percentage in the right superior quadrant in the frontal plane; Frontal quadrant IV, QRS loop percentage in the left superior quadrant in the frontal plane;

Horizontal quadrant I, QRS loop percentage in the left anterior quadrant in the horizontal plane; Horizontal quadrant II, QRS loop percentage in the right anterior quadrant in the horizontal plane; Horizontal quadrant III, QRS loop percentage in the right anterior quadrant in the horizontal plane; Horizontal quadrant IV, QRS loop percentage in the left anterior quadrant in the horizontal plane;

Sagittal quadrant I, QRS loop percentage in the anterior inferior quadrant in the sagittal plane; Sagittal quadrant II, QRS loop percentage in the posterior inferior quadrant in the sagittal plane; Sagittal quadrant III, QRS loop percentage in the posterior superior quadrant in the sagittal plane; Sagittal quadrant IV, QRS loop percentage in the anterior superior quadrant in the sagittal plane;

*significant, p<0.05

Supplementary table 3. The difference of VCG findings between normal children and DMD patients aged from 7 to 8 years old.

|  | Normal children (n=24) | DMD patients (n=117) | P value |
| --- | --- | --- | --- |
| Age(years) | 8.19±0.62 | 8.03±0.58 | 0.213 |
| Heart rate (bpm) | 75.59±9.58 | 87.96±13.13 | <0.001* |
| Scoliosis, n (%) | 0, (0%) | 0, (0%) | - |
| Loss of ambulation, n (%) | 0, (0%) | 0, (0%) | - |
| FVC%<80%, n (%) | 0, (0%) | 0, (0%) | - |
| Corticosteroids, n (%) | 0, (0%) | 115, (98.3%) | - |
| **Cardiac Treatment** |  |  | - |
| ACE inhibitor, n (%) | 0, (0%) | 9, (8.00%) | - |
| b-blocker, n (%) | 0, (0%) | 8, (7.20%) | - |
| ACE inhibitor+b-blocker, n (%) | 0, (0%) | 2, (1.70%) | - |
| **ECG parameters** |  |  |  |
| P-wave axis(°) | 30.96±26.17 | 44.79±20.90 | 0.004* |
| QRS-wave axis(°) | 35.89±13.60 | 53.51±24.11 | <0.001* |
| T -wave axis(°) | 64.30±23.34 | 56.87±20.76 | 0.104 |
| QT(ms) | 373.26±20.30 | 344.32±24.68 | <0.001* |
| QTc(ms) | 417.11±18.97 | 413.96±18.84 | 0.435 |
| P wave amplitude | 7.19±25.69 | 1.58±11.36 | 0.085 |
| RV1 in amplitude | 0.58±0.36 | 1.28±0.52 | <0.001* |
| RV5 in amplitude | 1.79±0.54 | 2.37±0.77 | <0.001* |
| SV1 in amplitude | 1.01±0.45 | 1.26±0.91 | 0.173 |
| R/S ratio in lead V1 | 0.63±0.41 | 1.41±0.76 | <0.001* |
| RV1+SV5 amplitude | 2.80±0.72 | 3.63±1.23 | 0.001* |
| Pathological Q waves in lead II | 0 | 1/117(0.86%) | - |
| **VCG parameters** |  |  |  |
| Frontal QRS Maximum Magnitude | 3.83±1.46 | 5.23±2.39 | 0.004* |
| Horizal QRS Maximum Magnitude | 4.08±1.46 | 5.10±2.15 | 0.023* |
| Sagittal QRS Maximum Magnitude | 5.13±2.53 | 6.16±2.68 | 0.071 |
| Frontal quadrant I (%) | 80.60±12.60 | 79.15±13.80 | 0.617 |
| Frontal quadrant II (%) | 7.11±7.31 | 9.38±7.67 | 0.164 |
| Frontal quadrant III (%) | 8.58±10.38 | 9.72±10.13 | 0.599 |
| Frontal quadrant IV (%) | 3.72±7.18 | 1.76±4.67 | 0.082 |
| Horizontal quadrant I (%) | 30.49±19.10 | 37.77±15.67 | 0.039* |
| Horizontal quadrant II (%) | 1.92±2.57 | 6.48±4.46 | <0.001* |
| Horizontal quadrant III (%) | 6.49±5.49 | 5.22±4.00 | 0.169 |
| Horizontal quadrant IV (%) | 61.10±18.49 | 50.54±17.11 | 0.005* |
| Sagittal quadrant I (%) | 29.34±20.85 | 40.22±16.49 | 0.004* |
| Sagittal quadrant II (%) | 60.06±21.13 | 49.12±19.57 | 0.011* |
| Sagittal quadrant III (%) | 8.76±8.81 | 6.18±10.70 | 0.247 |
| Sagittal quadrant IV (%) | 1.86±2.18 | 4.65±6.92 | 0.041* |
| Frontal QRS-T angle (°) | 12.94±27.89 | 13.80±25.72 | 0.878 |
| Horizontal QRS-T angle (°) | 33.48±32.48 | 33.07±30.19 | 0.949 |
| Sagittal QRS-T angle (°) | 55.69±46.75 | 75.54±82.56 | 0.094 |

The data are presented as the mean ± SD for continuous variables and as the percentage for the categorical variables.

Frontal quadrant I, QRS loop percentage in the left inferior quadrant in the frontal plane; Frontal quadrant II, QRS loop percentage in the right inferior quadrant in the frontal plane; Frontal quadrant III, QRS loop percentage in the right superior quadrant in the frontal plane; Frontal quadrant IV, QRS loop percentage in the left superior quadrant in the frontal plane;

Horizontal quadrant I, QRS loop percentage in the left anterior quadrant in the horizontal plane; Horizontal quadrant II, QRS loop percentage in the right anterior quadrant in the horizontal plane; Horizontal quadrant III, QRS loop percentage in the right anterior quadrant in the horizontal plane; Horizontal quadrant IV, QRS loop percentage in the left anterior quadrant in the horizontal plane;

Sagittal quadrant I, QRS loop percentage in the anterior inferior quadrant in the sagittal plane; Sagittal quadrant II, QRS loop percentage in the posterior inferior quadrant in the sagittal plane; Sagittal quadrant III, QRS loop percentage in the posterior superior quadrant in the sagittal plane; Sagittal quadrant IV, QRS loop percentage in the anterior superior quadrant in the sagittal plane;

*significant, p<0.05

Supplementary table 4. The difference of VCG findings between normal children and DMD patients aged from 9 to 12 years old.

|  | Normal children (n=22) | DMD patients (n=157) | P value |
| --- | --- | --- | --- |
| Age(years) | 10.59±0.73 | 10.58±1.03 | 0.951 |
| Heart rate (bpm) | 77.64±8.26 | 86.66±14.15 | <0.001* |
| Scoliosis, n (%) | 0, (0%) | 0, (0%) | - |
| Loss of ambulation, n (%) | 0, (0%) | 0, (0%) | - |
| FVC%<80%, n (%) | 0, (0%) | 0, (0%) | - |
| Corticosteroids, n (%) | 0, (0%) | 155, (98.7%) | - |
| **Cardiac Treatment** |  |  | - |
| ACE inhibitor, n (%) | 0, (0%) | 17, (10.8%) | - |
| b-blocker, n (%) | 0, (0%) | 15, (9.55%) | - |
| ACE inhibitor+b-blocker, n (%) | 0, (0%) | 9, (5.70%) | - |
| **ECG parameters** |  |  |  |
| P-wave axis(°) | 42.73±18.56 | 49.64±24.18 | 0.199 |
| QRS-wave axis(°) | 42.45±10.55 | 59.31±29.60 | <0.001* |
| T -wave axis(°) | 67.68±18.34 | 58.06±26.11 | 0.037* |
| QT(ms) | 365.55±19.21 | 345.68±30.23 | 0.003* |
| QTc(ms) | 414.73±20.97 | 410.43±36.76 | 0.593 |
| P wave amplitude | 0.09±0.05 | 2.37±28.40 | 0.708 |
| RV1 in amplitude | 0.52±0.28 | 1.25±0.53 | <0.001* |
| RV5 in amplitude | 1.77±0.59 | 2.37±0.86 | 0.002* |
| SV1 in amplitude | 1.00±0.47 | 0.99±0.54 | 0.913 |
| R/S ratio in V1 | 0.69±0.50 | 1.72±1.69 | <0.001* |
| RV1+SV5 | 2.77±0.76 | 3.35±1.16 | 0.003* |
| Pathological Q waves in lead II | 0 | 6/157(3.8%) | - |
| **VCG parameters** |  |  |  |
| Frontal QRS Maximum Magnitude | 2.77±0.76 | 3.35±1.16 | <0.001* |
| Horizal QRS Maximum Magnitude | 0.52±0.28 | 1.25±0.53 | 0.004* |
| Sagittal QRS Maximum Magnitude | 4.48±1.61 | 6.58±2.89 | <0.001* |
| Frontal quadrant I (%) | 83.14±15.91 | 74.57±19.15 | 0.047* |
| Frontal quadrant II (%) | 4.30±5.21 | 11.05±9.66 | <0.001* |
| Frontal quadrant III (%) | 9.27±12.26 | 11.89±14.53 | 0.421 |
| Frontal quadrant IV (%) | 3.30±6.25 | 2.40±7.46 | 0.590 |
| Horizontal quadrant I (%) | 26.22±16.91 | 40.43±16.98 | <0.001* |
| Horizontal quadrant II (%) | 2.09±2.42 | 10.20±11.90 | <0.001* |
| Horizontal quadrant III (%) | 5.16±4.29 | 5.05±4.30 | 0.910 |
| Horizontal quadrant IV (%) | 66.53±18.60 | 44.75±19.97 | <0.001* |
| Sagittal quadrant I (%) | 27.18±18.53 | 43.58±18.13 | <0.001* |
| Sagittal quadrant II (%) | 65.57±22.58 | 44.53±19.70 | <0.001* |
| Sagittal quadrant III (%) | 6.12±7.30 | 4.92±6.94 | 0.451 |
| Sagittal quadrant IV (%) | 1.13±1.38 | 7.07±11.22 | 0.014* |
| Frontal QRS-T angle (°) | 9.18±6.49 | 18.46±34.06 | 0.205 |
| Horizontal QRS-T angle (°) | 29.27±28.88 | 38.50±32.43 | 0.207 |
| Sagittal QRS-T angle (°) | 43.68±37.52 | 57.57±56.35 | 0.139 |

The data are presented as the mean ± SD for continuous variables and as the percentage for the categorical variables.

Frontal quadrant I, QRS loop percentage in the left inferior quadrant in the frontal plane; Frontal quadrant II, QRS loop percentage in the right inferior quadrant in the frontal plane; Frontal quadrant III, QRS loop percentage in the right superior quadrant in the frontal plane; Frontal quadrant IV, QRS loop percentage in the left superior quadrant in the frontal plane;

Horizontal quadrant I, QRS loop percentage in the left anterior quadrant in the horizontal plane; Horizontal quadrant II, QRS loop percentage in the right anterior quadrant in the horizontal plane; Horizontal quadrant III, QRS loop percentage in the right anterior quadrant in the horizontal plane; Horizontal quadrant IV, QRS loop percentage in the left anterior quadrant in the horizontal plane;

Sagittal quadrant I, QRS loop percentage in the anterior inferior quadrant in the sagittal plane; Sagittal quadrant II, QRS loop percentage in the posterior inferior quadrant in the sagittal plane; Sagittal quadrant III, QRS loop percentage in the posterior superior quadrant in the sagittal plane; Sagittal quadrant IV, QRS loop percentage in the anterior superior quadrant in the sagittal plane;

*significant, p<0.05

Supplementary table 5. The difference of VCG findings between normal children and DMD patients aged equal or higher than 13 years old.

|  | Normal children (n=20) | DMD patients (n=40) | P value |
| --- | --- | --- | --- |
| Age(years) | 14.50±1.47 | 14.88±1.20 | 0.286 |
| Heart rate (bpm) | 73.80±19.38 | 82.85±12.31 | 0.032* |
| Scoliosis, n (%) | 0, (0%) | 0, (0%) | - |
| Loss of ambulation, n (%) | 0, (0%) | 0, (0%) | - |
| FVC%<80%, n (%) | 0, (0%) | 0, (0%) | - |
| Corticosteroids, n (%) | 0, (0%) | 40, (100%) | - |
| **Cardiac Treatment** |  |  | - |
| ACE inhibitor, n (%) | 0, (0%) | 15, (37.5%) | - |
| b-blocker, n (%) | 0, (0%) | 14, (35.0%) | - |
| ACE inhibitor+b-blocker, n (%) | 0, (0%) | 10, (25.0%) | - |
| **ECG parameters** |  |  |  |
| P-wave axis(°) | 42.50±34.75 | 54.45±24.91 | 0.131 |
| QRS-wave axis(°) | 42.30±19.69 | 64.50±34.84 | 0.011* |
| T -wave axis(°) | 70.10±24.72 | 70.83±31.12 | 0.928 |
| QT(ms) | 377.20±23.70 | 351.65±24.95 | <0.001* |
| QTc(ms) | 405.40±16.99 | 410.78±24.99 | 0.390 |
| P wave amplitude | 93.30±7.52 | 89.80±10.37 | 0.346 |
| RVI in amplitude | 0.51±0.39 | 1.25±0.77 | <0.001* |
| RV5 in amplitude | 1.79±0.55 | 2.11±0.94 | 0.102 |
| SV1 in amplitude | 1.00±0.51 | 0.71±0.56 | 0.061 |
| R/S ratio in V1 | 0.53±0.35 | 3.41±5.25 | 0.002* |
| RV5+SV1 | 2.79±0.77 | 2.82±1.38 | 0.900 |
| Pathological Q waves in lead II | 0 | 3/40(7.5%) | - |
| **VCG parameters** |  |  |  |
| Frontal QRS Maximum Magnitude | 4.43±1.80 | 5.89±4.42 | 0.161 |
| Horizal QRS Maximum Magnitude | 4.08±1.65 | 3.91±1.67 | 0.701 |
| Sagittal QRS Maximum Magnitude | 4.65±1.66 | 4.97±3.28 | 0.680 |
| Frontal quadrant I (%) | 81.06±17.09 | 65.39±27.74 | 0.009* |
| Frontal quadrant II (%) | 5.87±8.92 | 15.71±16.16 | 0.004* |
| Frontal quadrant III (%) | 10.82±16.39 | 17.13±21.25 | 0.249 |
| Frontal quadrant IV (%) | 2.25±4.63 | 1.84±3.41 | 0.700 |
| Horizontal quadrant I (%) | 24.02±18.66 | 45.97±24.40 | <0.001* |
| Horizontal quadrant II (%) | 2.24±3.93 | 10.00±11.14 | <0.001* |
| Horizontal quadrant III (%) | 10.85±13.76 | 8.65±10.35 | 0.490 |
| Horizontal quadrant IV (%) | 62.89±24.60 | 35.38±23.34 | <0.001* |
| Sagittal quadrant I (%) | 22.75±16.35 | 47.56±26.07 | <0.001* |
| Sagittal quadrant II (%) | 63.85±27.10 | 39.05±23.20 | <0.001* |
| Sagittal quadrant III (%) | 9.89±15.58 | 6.67±10.04 | 0.336 |
| Sagittal quadrant IV (%) | 3.51±10.43 | 6.98±6.90 | 0.129 |
| Frontal QRS-T angle (°) | 6.50±6.18 | 33.00±50.81 | 0.002* |
| Horizontal QRS-T angle (°) | 52.25±42.71 | 59.59±39.41 | 0.491 |
| Sagittal QRS-T angle (°) | 110.30±81.07 | 79.68±60.10 | 0.104 |

The data are presented as the mean ± SD for continuous variables and as the percentage for the categorical variables.

Frontal quadrant I, QRS loop percentage in the left inferior quadrant in the frontal plane; Frontal quadrant II, QRS loop percentage in the right inferior quadrant in the frontal plane; Frontal quadrant III, QRS loop percentage in the right superior quadrant in the frontal plane; Frontal quadrant IV, QRS loop percentage in the left superior quadrant in the frontal plane;

Horizontal quadrant I, QRS loop percentage in the left anterior quadrant in the horizontal plane; Horizontal quadrant II, QRS loop percentage in the right anterior quadrant in the horizontal plane; Horizontal quadrant III, QRS loop percentage in the right anterior quadrant in the horizontal plane; Horizontal quadrant IV, QRS loop percentage in the left anterior quadrant in the horizontal plane;

Sagittal quadrant I, QRS loop percentage in the anterior inferior quadrant in the sagittal plane; Sagittal quadrant II, QRS loop percentage in the posterior inferior quadrant in the sagittal plane; Sagittal quadrant III, QRS loop percentage in the posterior superior quadrant in the sagittal plane; Sagittal quadrant IV, QRS loop percentage in the anterior superior quadrant in the sagittal plane;

*significant, p<0.05

Supplementary Table 6. Demographic and VCG findings between positive LGE and negative LGE patient groups in DMD

|  | LGE+ (n=17) | LGE- (n=44) | P value |
| --- | --- | --- | --- |
| Age(years) | 9.84±2.92 | 9.18±1.85 | 0.296 |
| Heart rate (bpm) | 87.71±14.13 | 91.00±13.89 | 0.412 |
| Height (cm) | 128.79±3.63 | 128.83±3.81 | 0.98 |
| Weight (kg) | 31.18±2.46 | 34.95±2.85 | 0.21 |
| Systolic blood pressure (mmHg) | 104.59±4.81 | 106.01±2.78 | 0.63 |
| Diastolic blood pressure (mmHg) | 66.00±5.91 | 68.03±2.20 | 0.44 |
| Scoliosis, n (%) | 1, (5.9%) | 0, (0.0%) | 0.279 |
| Loss of ambulation, n (%) | 0, (0.0%) | 0, (0.0%) | - |
| **Respiratory Function** |  |  |  |
| FVC%<80%, n (%) | 0, (0.0%) | 0, (0.0%) | - |
| **Steriod Treatment** |  |  |  |
| Corticosteroids, n (%) | 16, (94.1%) | 39, (88.6%) | 0.460 |
| Time to initiate steriods (years) | 6.18±1.62 | 6.95±1.92 | 0.170 |
| Time from initiating steriods (months) | 36.94±36.31 | 23.23±20.90 | 0.158 |
| **Cardiac Treatment** |  |  | 0.885 |
| ACE inhibitor, n (%) | 4, (23.5%) | 14, (31.8%) |  |
| b-blocker, n (%) | 3, (17.6%) | 9, (20.5%) |  |
| ACE inhibitor+b-blocker, n (%) | 5, (29.4%) | 10, (22.7%) |  |
| **ECG findings** |  |  |  |
| P-wave axis(°) | 49.00±26.76 | 54.05±25.01 | 0.491 |
| QRS-axis(°) | 49.94±19.16 | 49.91±22.86 | 0.996 |
| T -wave axis(°) | 69.59±19.82 | 62.18±19.18 | 0.185 |
| QT(ms) | 345.65±26.30 | 340.36±24.57 | 0.463 |
| QTc(ms) | 414.53±17.92 | 416.16±15.70 | 0.728 |
| P wave amplitude | 0.09±0.03 | 0.10±0.04 | 0.149 |
| RV5 in amplitude | 2.29±0.74 | 2.55±0.84 | 0.268 |
| SV1 in amplitude | 1.22±0.46 | 0.98±0.51 | 0.092 |
| R/S ratio in V1 | 1.41±1.39 | 1.92±1.68 | 0.479 |
| RV1+SV5 amplitude | 1.33±0.58 | 1.24±0.72 | 0.639 |
| Pathological Q waves in lead II | 1, (5.9%) | 0, (0.0%) | 0.279 |
| **VCG findings** |  |  |  |
| Frontal QRS Maximum Magnitude | 4.95±1.36 | 5.21±3.31 | 0.762 |
| Horizal QRS Maximum Magnitude | 4.59±1.47 | 5.07±3.28 | 0.562 |
| Sagittal QRS Maximum Magnitude | 5.91±1.76 | 6.37±2.94 | 0.552 |
| Frontal quadrant I (%) | 77.81±17.84 | 75.66±15.67 | 0.645 |
| Frontal quadrant II (%) | 7.71±5.70 | 11.16±8.57 | 0.132 |
| Frontal quadrant III (%) | 12.02±13.70 | 11.28±12.98 | 0.845 |
| Frontal quadrant IV (%) | 2.46±5.95 | 1.89±4.79 | 0.702 |
| Horizontal quadrant I (%) | 40.42±16.20 | 42.72±19.59 | 0.677 |
| Horizontal quadrant II (%) | 7.59±5.81 | 6.57±4.43 | 0.464 |
| Horizontal quadrant III (%) | 3.63±3.38 | 6.01±4.75 | 0.064 |
| Horizontal quadrant IV (%) | 48.30±20.98 | 44.92±20.20 | 0.565 |
| Sagittal quadrant I (%) | 43.31±16.18 | 42.90±17.04 | 0.932 |
| Sagittal quadrant II (%) | 44.99±21.55 | 45.19±20.81 | 0.974 |
| Sagittal quadrant III (%) | 4.66±3.50 | 4.56±5.24 | 0.910 |
| Sagittal quadrant IV (%) | 4.66±3.50 | 4.56±5.24 | 0.941 |
| Frontal QRS-T angle (°) | 14.12±11.83 | 12.14±11.07 | 0.541 |
| Horizontal QRS-T angle (°) | 27.47±23.46 | 30.61±24.24 | 0.649 |
| Sagittal QRS-T angle (°) | 40.94±37.85 | 53.75±51.87 | 0.359 |

The data are presented as the mean ± SD for continuous variables and as the percentage for the categorical variables.

Frontal quadrant I, QRS loop percentage in the left inferior quadrant in the frontal plane; Frontal quadrant II, QRS loop percentage in the right inferior quadrant in the frontal plane; Frontal quadrant III, QRS loop percentage in the right superior quadrant in the frontal plane; Frontal quadrant IV, QRS loop percentage in the left superior quadrant in the frontal plane;

Horizontal quadrant I, QRS loop percentage in the left anterior quadrant in the horizontal plane; Horizontal quadrant II, QRS loop percentage in the right anterior quadrant in the horizontal plane; Horizontal quadrant III, QRS loop percentage in the right anterior quadrant in the horizontal plane; Horizontal quadrant IV, QRS loop percentage in the left anterior quadrant in the horizontal plane;

Sagittal quadrant I, QRS loop percentage in the anterior inferior quadrant in the sagittal plane; Sagittal quadrant II, QRS loop percentage in the posterior inferior quadrant in the sagittal plane; Sagittal quadrant III, QRS loop percentage in the posterior superior quadrant in the sagittal plane; Sagittal quadrant IV, QRS loop percentage in the anterior superior quadrant in the sagittal plane;

*Statistically significant (P<0.05)
